# Combination of first-line chemotherapy with Kanglaite injections versus first-line chemotherapy alone for advanced non-small-cell lung cancer: study protocol for an investigator-initiated, multicenter, open-label, randomized controlled trial

**DOI:** 10.1186/s13063-021-05169-w

**Published:** 2021-03-17

**Authors:** Ruike Gao, Ying Zhang, Wei Hou, Jie Li, Guanghui Zhu, Xiaoxiao Zhang, Bowen Xu, Zhe Wu, Heping Wang

**Affiliations:** 1 Guang’anmen Hospital, China Academy of Chinese Medical Sciences, Beijing, China; 2 China Academy of Chinese Medical Sciences, Beijing, China; 3 Beijing University of Chinese Medicine, Beijing, China

**Keywords:** Kanglaite injection, Non-small cell lung cancer, Chinese herbal medicine, Randomized controlled trial, Progression-free survival, Protocol

## Abstract

**Background:**

Non-small-cell lung cancer (NSCLC) is usually diagnosed at an advanced stage, and chemotherapy is the main treatment for this disease. Kanglaite injections (KLTi) have been widely used for the treatment of cancer in China. KLTi combined with chemotherapy could improve the short-term efficacy, quality of life, and performance status for NSCLC compared with chemotherapy alone. This trial aims to assess the long-term efficacy and safety of KLTi in combination with chemotherapy for the treatment of advanced NSCLC.

**Methods:**

This will be an investigator-initiated multicenter open-label randomized controlled trial. We will randomly assign 334 eligible participants with stage IIIA-IV NSCLC to the treatment or control groups in a 1:1 ratio. Patients in both groups will be administered 4–6 cycles of first-line platinum-based double chemotherapy regimens. Patients with complete response, partial response, or stable disease after 4–6 cycles will receive non-platinum single-agent chemotherapy. Patients in the treatment group are to receive intravenous KLTi 200 ml per day continuously for 14 days, commencing on the first day of chemotherapy. The treatment will be discontinued at the time of disease progression or until unacceptable toxicity is noted. The follow-up will be conducted every 2 months until death, loss of follow-up, or 12 months from randomized enrollment. The primary outcome will be progression-free survival (PFS). The secondary outcomes will be the objective response rate, 1-year survival rate, quality of life, living ability, and blood lipids. The safety outcome will be the rate of adverse events.

**Discussion:**

This study will be the first randomized controlled trial in which PFS is used as the primary outcome to test whether KLTi combined with first-line chemotherapy has superior efficacy and reduced toxicity compared to chemotherapy alone in advanced NSCLC. This will also be the first clinical study to observe the effects of KLTi on blood lipids.

**Trial registration:**

ClinicalTrials.gov NCT03986528. Prospectively registered on 30 May 2019.

## Background

Lung cancer is the most commonly diagnosed cancer and the leading cause of cancer-related deaths worldwide [1]. Non-small cell lung cancer (NSCLC) accounts for > 80% of lung cancer cases and is usually diagnosed at an advanced stage, making it ineligible for a curative treatment [2]. Over the last decade, the advent of molecular therapies targeting genomic addictive abnormalities and immune checkpoint inhibitors (ICI) has resulted in encouraging progress in the treatment of advanced/metastatic NSCLC [3–7]. However, due to development of resistance to molecular targeted drugs, patients eventually experience disease progression [8, 9]. Indeed, the administration of anti-PD-(L)1 antibodies as ICIs is associated with drug resistance, pseudo-progression, hyper-progression, mixed progression, and immune-related adverse events [10].

Systemic chemotherapy remains an important component of the advanced NSCLC treatment. Patients with negative genetic mutations are routinely treated with cisplatin-based combination chemotherapy [11, 12]. A combination of chemotherapy and molecular targeted drugs showed improved efficacy in NSCLC patients with certain genetic mutations [13–15]. Specifically, cisplatin-based combination chemotherapy regimen has been used as a standard treatment after the failure of targeted therapy and prolongs survival in NSCLC patients [16, 17]. Furthermore, chemotherapy combined with ICIs showed encouraging antitumor results [18, 19]. However, most chemotherapeutic agents have severe AEs that drastically affect the patient’s quality of life. Moreover, the use of chemotherapeutic agents is associated with drug resistance [20]. Therefore, there is an urgent need for new improved treatment strategies.

Traditional Chinese medicine (TCM) has been used to reduce the side effects of cancer chemotherapy and improve the effects of treatment. Previous findings have shown that TCM, combined with chemotherapy prolonged the survival in patients with advanced NSCLC, improved patient symptoms and reduced chemotherapy-related side effects [21–23]. Kanglaite injection (KLTi), an intravenous TCM modality, has been used to treat millions of patients with cancer since 1997 [24, 25]. KLTi is a microemulsion of Coix seed oil extracted from Semen Coicis, a traditional Chinese medicinal plant, which has demonstrated immunomodulatory effects and antitumor activities [24, 26]. Previous studies have shown that KLTi promoted tumor cell apoptosis by upregulating the expression of the *p53* and *FAS* genes, as well as Caspase-3, proliferating-cell nuclear antigen (*PCNA*), and *p21WAFI/CIPI*, and by downregulating the expression of the cyclin A, cyclin E1, and cyclin F coding genes [27]. It can also suppress tumor growth by inhibiting the activity of protein kinase C and NF-κB signaling [28]. Moreover, some studies have demonstrated that KLTi had antitumor and immunostimulatory activities in C57BL/6 mice with Lewis lung carcinoma [29]. A recent meta-analysis found that KLTi in combination with chemotherapy improved clinical efficacy, quality of life, and reduced the incidence of AEs compared with chemotherapy alone in patients with advanced NSCLC [30–33]. However, previous research has mainly focused on short-term efficacy and toxicity, and there is a lack of data on long-term survival outcomes. The use of Coix seed oil is associated with a reduction in blood lipids [34]. KLTi can significantly suppress proliferation and induce apoptosis and differentiation in 3T3-L1 preadipocyte cells without affecting food intake [26]. However, there is no clinical data to evaluate the effect of KLTi on lipid profiles.

Therefore, this study aimed to investigate the long-term efficacy and safety of KLTi combined with first-line chemotherapy in advanced NSCLC. We also sought to obtain clinical evidence to determine the effect of KLTi on lipid profiles.

## Method/design

### Study design

**Fig. 1:**
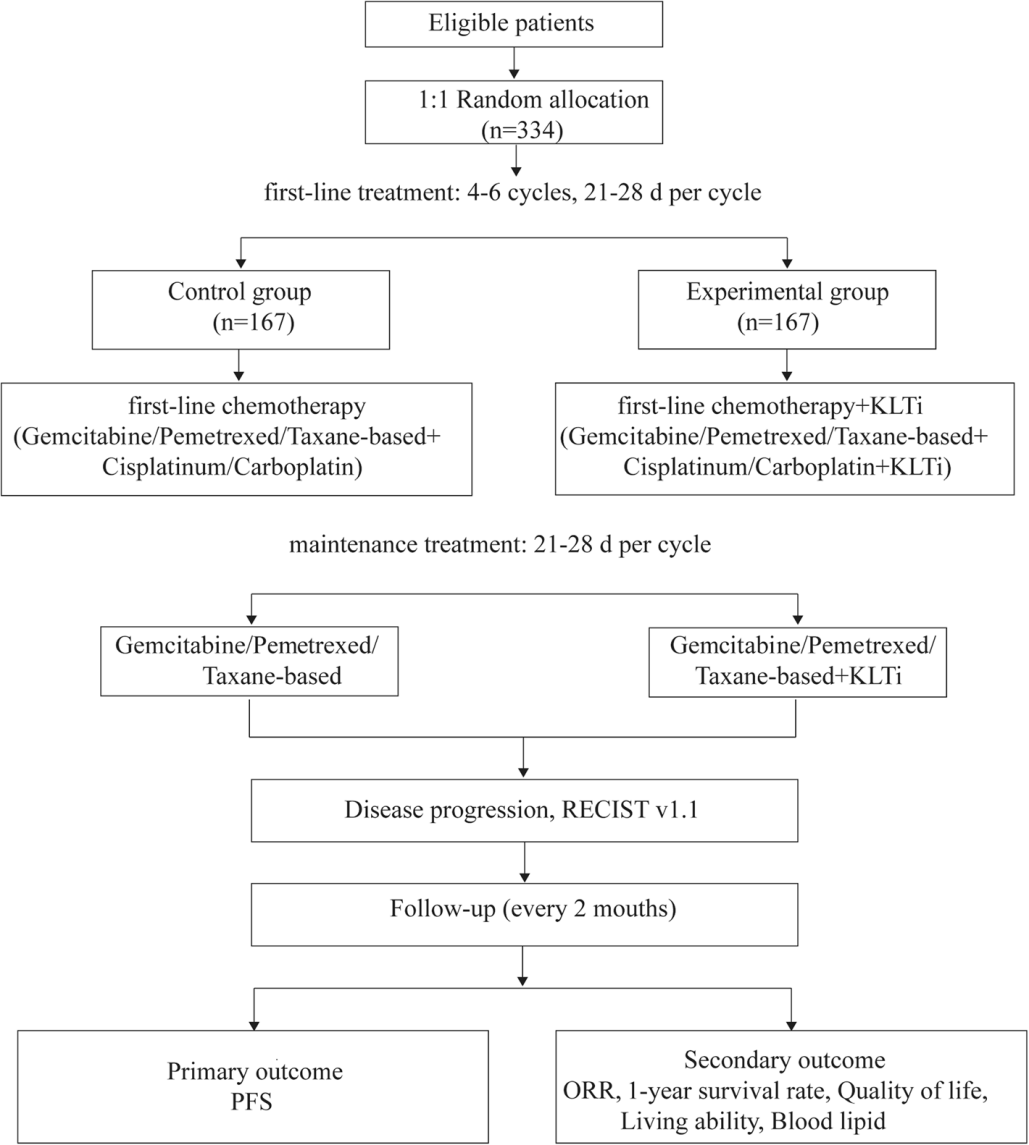
Flow chart of the study. KLTi, Kanglaite injection; PFS, progression-free survival; ORR, objective response rate

This study is a multicenter, randomized, open-label clinical trial with two parallel arms. The purpose is to estimate the effectiveness and safety of KLTi in patients with advanced NSCLC who have not received anticancer treatment prior to participation in this study. Eligible participants will be randomly assigned to either the treatment or control group using an online tool. Patients in the treatment group will receive KLTi combined with first-line chemotherapy and those in the control group will receive first-line chemotherapy alone. Eligible patients will receive therapy continuously until either disease progression according to the Response Evaluation Criteria in Solid Tumors 1.1 (RECIST 1.1) [35], unacceptable toxicity, completion of 12 months starting from the time of randomized enrollment, or withdrawal of informed consent. After disease progression, follow-up will be conducted every 2 months until death, loss of follow-up, or 12 months from randomized enrollment. The flow chart of the study design is shown in Fig. 1. This protocol has been registered on Clinical Trials (ClinicalTrials.gov, ID: NCT03986528).

### Eligibility criteria

The inclusion criteria will be as follows:

1. A histologically or cytologically confirmed diagnosis of Stage III-IV NSCLC, with no history of anticancer treatment including chemotherapy;
2. Male or female aged 18–75 years;
3. Eastern Cooperative Oncology Group (ECOG) performance score 0–2;
4. Life expectancy of at least 3 months;
5. At least one radiographically measurable lesion as per RECIST 1.1;
6. Willing to join the clinic trial and sign the informed consent;
7. Able to comply with scheduled visits and treatments.

The exclusion criteria will be as follows:

1. Presence of cerebral metastases;
2. Confirmed positive for expression of epidermal growth factor receptor (*EGFR*), activin receptor-like kinase (*ALK*), c-ros oncogene 1 (*ROS1*) mutation, or programmed death-ligand 1 (PD-L1) (tumor proportion score [TPS] ≥ 50%) in a genetic test;
3. Participants with malignant pleural effusion underwent intrapleural injection chemotherapy;
4. Currently undergoing or preparing for treatment with target therapy;
5. Currently undergoing or preparing for radiotherapy treatment to the thorax;
6. Currently undergoing or preparing for treatment with tumor immunotherapy;
7. Currently undergoing lipid-decreasing treatment;
8. Pregnant or breastfeeding women;
9. Fertile patients who are unwilling or unable to take effective contraceptive measures during the research period until 6 months after the study end date;
10. A history of mental disorders;
11. Severe and uncontrolled organic lesion or infection, including but not limited to cardiopulmonary failure and renal failure, which lead to poor tolerance of chemotherapy;
12. Participated in other clinical trials of small molecule research drugs within 28 days prior to enrollment, or participated in other clinical trials of large molecule research drugs within 3 months before enrollment;
13. Known allergy or intolerance to study medications;
14. Considered to be otherwise unsuitable for the clinical study by researchers.

### Interventions

Eligible patients will be randomized 1:1 to the treatment or control group. Patients in both groups will receive 4–6 cycles of first-line platinum-based double chemotherapy regimens. Patients with complete response (CR), partial response (PR), or stable disease after 4–6 cycles will receive non-platinum single-agent chemotherapy. The chemotherapy regimens are in accordance with the National Comprehensive Cancer Network (NCCN) guidelines (2019.V3) [36] (Table 1). Patients in the treatment group are to receive intravenous KLTi 200 ml per day continuously for 14 days, commencing on the first day of chemotherapy. In this study, treatment will be discontinued if one or more of the following occurs: (1) unacceptable toxicity; (2) withdrawal of informed consent; (3) tumor progression; or (4) completion of 12 months starting from the time of randomized enrollment. Treatment for other conditions, such as hypertension, diabetes mellitus, and other chronic diseases, will be permitted during protocol treatment. However, anti-angiogenic agents, other TCM injections, Chinese patent medicines with antitumor effects, and other antitumor methods are prohibited for all patients during the treatment course.

**Table 1:** Chemotherapy regimens for NSCLC

Pathologic types	Chemotherapy regimen	Dosage (mg/m^2^)	Medication time
Squamous cell carcinoma	GC		
	Gemcitabine	1000	D1, 8
	Cisplatinum	75	D1–3
	GC		
	Gemcitabine	1000	D1, 8
	Carboplatin	AUC = 6	D1
Adenocarcinoma, large cell, NSCLC not otherwise specified (NOS)	PP		
	Pemetrexed	500	D1
	Cisplatinum	75	D1–3
	PC		
	Pemetrexed	500	D1
	Carboplatin	AUC = 6	D1

Notes: If patients experience gemcitabine-associated intolerable toxicities, paclitaxel will be used instead of gemcitabine. The dose of paclitaxel is 135 mg/m^2^ D1. The dosages of the chemotherapy regimens are provided only for reference, and specific implementation will take clinical application as a standard

*AUC* area under the curve, *NSCLC* non-small cell lung cancer

KLTi (specifications: 100 ml/tube) will be manufactured by Zhejiang Kanglaite Pharmaceutical Co. Ltd. (Hangzhou, China). Tumor response assessment will be performed every 6–8 weeks during protocol treatment using the RECIST guidelines (version 1.1) [35].

### Outcome measures

#### Primary outcome

##### Progression-free survival (PFS)

The primary outcome is PFS, which will be measured every 41–56 days (2 cycles) from the randomization to the onset of disease progression or death, whichever comes first. Patients without progression at the cutoff date will be censored on the date of the last contact.

#### Secondary outcomes

##### Objective response rate (ORR)

The ORR is the percentage of participants who have a complete response (CR), defined as the disappearance of all target lesions or a partial response (PR), defined as ≥ 30% decrease in the sum of the diameters of target lesions as assessed by RECIST 1.1 [35]. This will be evaluated every 41–56 days (2 cycles) until disease progression, death, or 12 months after randomized enrollment.

##### One-year survival rate

The 1-year survival rate refers to the proportion of patients with a survival period of more than 1 year starting from randomized enrollment.

##### Patient quality of life

Quality of life will be assessed based on the European Organization for Research and Treatment of Cancer (EORTC) Quality of Life Questionnaire Core 30 (QLQ-C30, version 3) [37, 38] and the Lung Cancer Symptom Scale (LCSS) [39] before and after each cycle of treatment for up to 12 months after randomized enrollment.

##### Living ability of the patient

The living ability of the patient will be evaluated according to the ECOG [40] and Karnofsky performance status (KPS) [41] before and after each cycle of treatment for up to 12 months after randomized enrollment.

##### Blood lipid profile

The total cholesterol (TC), triglyceride (TG), high-density lipoprotein cholesterol (HDL-C), and low-density lipoprotein cholesterol (LDL-C) will be measured to evaluate the effect of KLTi on serum lipids in this study. Levels will be measured every 41–56 days (2 cycles) until disease progression, death, or 12 months after randomized enrollment.

### Safety outcomes

Patients will be monitored weekly for AEs, based on the National Cancer Institute’s Common Terminology Criteria for Adverse Events v.4.03 (CTCAE v.4.03), from baseline to disease progression, death, or 12 months after randomized enrollment. If any AEs occur, they will be recorded in a timely manner and the physician will treat the patient according to the actual situation. Serious AEs will be reported to the ethics committee. Any AEs caused by the study will be reimbursed by insurance.

### Sample size

The sample size was calculated based on the primary study outcome. The previously reported PFS of maintenance pemetrexed plus best supportive care for NSCLC was 4.3 months (95% confidence interval [CI]: 4.1–4.7), whereas the PFS for placebo plus best supportive care was 2.6 months (95% CI: 1.7–2.8) [42]. Some studies showed that KLTi in combination with chemotherapy improved clinical efficacy compared with chemotherapy alone in patients with NSCLC [30–33]. Therefore, we predict that the PFS in the treated group will be 2.1 months longer than that in the control group. A sample size calculation using PASS software (Version 11.0, NCSS, LLC. Kaysville, UT, USA) estimates that 167 participants would be required in the treatment and control groups (i.e., total of *N* = 334), with 95% confidence level, 80% power, and 20% drop-out rate.

### Recruitment

Participants will be recruited from 18 upper first-class hospitals across 15 provinces in China (Table 2). In addition, the recruitment will be done through outpatient clinics, recruitment posters in the hospital, and website advertisements. The clinical research coordinator (CRC) will schedule eligible patients for screening by telephone or on-site. The principal investigator and co-investigators will recruit eligible participants among these patients and obtain their written informed consent at each site. If the patient is incapable of consent, informed consent will be obtained from an authorized surrogate. The planned recruitment period is 14 months from August 2019 to December 2020. Due to COVID-19, the recruitment is expected to end in December 2021. To achieve adequate participant enrollment, KLTi will be provided to participants free-of-charge.

**Table 2:** List of the participating hospitals

Number	Hospital name	Region (in China)
01	Guang’anmen Hospital, China Academy of Chinese Medical Science	Beijing
02	The First Affiliated Hospital of Liaoning University of Traditional Chinese Medicine	Shenyang
03	The First Affiliated Hospital of Guangzhou University of Chinese Medicine	Guangzhou
04	The Affiliated Hospital of Shandong University of Traditional Chinese Medicine	Jinan
05	Jiangsu Province Hospital of Chinese Medicine	Nanjing
06	Shanxi Provincial Cancer Hospital	Taiyuan
07	Tianjin Medical University Cancer Institute & Hospital	Tianjin
08	Hunan Provincial Tumor Hospital	Changsha
09	Hunan Academy of Traditional Chinese Medicine Affiliated Hospital	Changsha
10	Gansu Provincial Tumor Hospital	Lanzhou
11	The Fourth Military Medical University Tangdu Hospital	Xian
12	Anhui Chest Hospital	Hefei
13	Chongqing Cancer Hospital	Chongqing
14	Zhengzhou Cancer Hospital	Zhengzhou
15	The First Affiliated Hospital of Zhejiang Chinese Medicine University	Hangzhou
16	XuZhou Central Hospital	XuZhou
17	Longhua Hospital Shanghai University of Traditional Chinese Medicine	Shanghai
18	Yueyang Hospital of Integrated Traditional Chinese and Western Medicine, Shanghai University of Traditional Chinese Medicine	Shanghai

### Randomization

Random assignment of eligible patients using the minimization technique will be performed through a web-based central randomization system, which will be operated by one physician in each center. This system ensures the allocation sequence unpredictable. Patients will be assigned to either the treatment or the control group (1:1) according to gender (female vs. male), age (> 60 vs. ≤ 60 years old), adenocarcinoma (yes vs. no), and staging (stage III vs. IV).

### Data collection and management

The data of all participants including those who will be discontinued or deviated from intervention protocols will be collected according to study protocol. Frequent follow-up phone call will be conducted to promote participant retention.

The case report forms (CRFs) will be used to collect data. To ensure that all data recorded in the CRFs is consistent with the original material, a CRC will be assigned to assist physicians to document and manage all visits and examinations. To ensure the timeliness of the data, the CRFs must be completed within 3 days of each visit.

An online web-based electronic data capture system will also be used for data collection and management. Double data entry is employed and performed by two independent researchers. The modified data will be highlighted in red. Patients will be identified on the electronic CRF (e-CRF) using initials and a unique code.

Confidentiality procedures are outlined on the consent form. All CRFs will be kept in locked cabinets. Only the research team will have access to the data.

### Blood sample collection and management

Peripheral venous blood samples (10.5 ml) will be collected under fasting conditions before chemotherapy infusion at baseline and every 2 cycles for up to 12 months after randomized enrollment or until withdrawal of informed consent, tumor progression, or death. Each blood sample (10.5 ml) will be divided into three fractions: 2 ml blood collected in an EDTA tube, 6 ml blood collected in an EDTA tube to obtain plasma, and 2.5 ml blood collected in a PAXgene Blood RNA tube. All collected samples will be stored anonymously at − 80 ° in the Cancer Laboratory of Guang’anmen Hospital. A specially assigned person will be in charge of the management of blood samples. Informed consent from each participant will be obtained before blood collection. The blood samples will be analyzed for multi-omics analysis in the current trial and for future use in ancillary studies.

### Quality control

On-site training will be carried out before the initiation of the study. This will enable the study physicians, nurses, and quality control personnel to fully apprehend the process of the entire trial. Any questions will be answered in a timely manner by the central study team. When disease progression occurs, the doctor-in-charge and chief physician will evaluate patient conditions based on imaging studies and will fill out the assessment form.

Three levels of quality control will be performed: a data manager and research secretary for each participating unit (first-level), a CRC and clinical research associate (CRA) of Beijing Kangpaite Medical Science and Technology Development Co., Ltd. (second-level), and six supervisors from the central research group (third-level). Subjects will be enrolled after the doctor-in-charge, CRC, CRA, and a trained oncologist from the central research group check the patient information against the inclusion and exclusion criteria. During the study, the CRC, CRA, and the supervisors from the central research group will carry out regular site visits to review protocol compliance, conduct source data verification, and assess drug accountability and management and trial procedures. They will also ensure that the study is being conducted according to the relevant regulatory and protocol requirements. The supervisors from the central research group also will regularly monitor the quality of the data obtained by the electronic data capture system.

### Statistical analysis

An independent, professional statistician will process the data for the results and the AEs using SAS 9.2 statistical software. *P* values < 0.05 will be considered statistically significant.

Data will be cross-checked for outliers and missing values. Outliers will be removed after data analysis, and missing values will be handled using carry forward methodology or considered for drop-out after data analysis. The measurement data will be described by mean, standard deviation, median, minimum, and maximum. The enumeration data will be expressed in frequency counts and percentages. PFS is the primary outcome and will be analyzed using the Kaplan-Meier method and compared between the two groups using the log-rank test. A stratified Cox proportional-hazards model and Efron’s method of tie handling will be used to assess the magnitude of the difference between the trial groups. We will use the chi-square test to compare the 1-year survival rate and ORR between treatment and control groups. Changes in CHO, TG, HDL-C, LDL-C, and the questionnaire scores of EORTC QLQ-C30 and LCSS will be assessed using paired sample *t*-test, Wilcoxon rank-sum test, or independent *t*-test.

## Discussion

Kanglaite, a type of Chinese herb product extracted from the Coix seed, is a microemulsion and has been used as an antitumor drug in China for many years. This study aims to explore the long-term efficacy and safety of KLTi combined with first-line chemotherapy for the treatment of advanced NSCLC.

In the previous studies of KLTi on advanced NSCLC, the primary endpoint was ORR, which was evaluated after 2 or 4 treatment cycles. But it cannot fully determine the clinical benefits of the trial drug. Overall survival (OS) is the gold standard marker of efficacy for any cancer treatment [43]. However, taking OS as the primary endpoint prolongs the study observation period, and the results may easily be affected by cross-treatment and follow-up treatments. Therefore, determining a surrogate endpoint for OS has been the subject of much discussion in recent years. Guidelines from both the European Medicines Agency (EMEA) and the United States Food and Drug Administration (FDA) describe PFS as an endpoint that may be used to demonstrate clinical benefit [44]. In operable NSCLC (adjuvant trials) and locally advanced NSCLC (radiotherapy trials), Mauguen et al. reported a high correlation between DFS/PFS and OS at the patient and trial level [45]. In addition, the magnitude of advantage in tumor response is suggested to contribute to a better prediction of OS-HR based on PFS-HR in clinical trials in patients with advanced NSCLC [46]. Accordingly, PFS is the primary endpoint in this study.

Some studies have found that there was a strong link between serum lipids and morbidity and mortality in certain types of cancer, including gastric cancer, gastrointestinal malignancies, and prostate cancer [47–49]. Furthermore, a study of blood lipid profiles and lung cancer risk in a meta-analysis on prospective cohort studies showed that levels of both serum HDL-C and TC were significantly inversely correlated with the incidence of lung cancer. In contrast, serum TG levels showed a significant positive correlation with lung cancer incidence. Coix seed oil is known to reduce blood lipids [34], and KLTi has been associated with a decrease in the lung cancer-promoting effects of high-fat diet (HFD)-induced obesity [26]. Therefore, this study will monitor the blood lipid profiles, including TC, HDL-C, LDL-C, and TG, to explore the effect of KLTi on blood lipids.

The placebo of KLTi is difficult to access in China, and it is therefore impossible to blind the study. To reduce this bias, all of the participating hospitals are tertiary grade A hospitals in China, with the same level of diagnosis and treatment. In addition, the doctor-in-charge and a chief physician will evaluate the condition of each subject when the disease progresses. Finally, to guarantee the reliability of the results of this study, we will adopt quality control measures for four different aspects: (i) training of researchers before the start of the project, (ii) management of enrolled patients, (iii) perform three levels of quality control, and (iv) online and offline monitoring of data.

We expect that this trial will provide a high degree of evidence for the efficacy and safety of KLTi combined with chemotherapy in treating advanced NSCLC. In addition, the blood lipid results will allow us to assess the effects of KLTi on blood lipids.

## Trial status

The trial was prospectively registered at ClinicalTrials.gov (ID: NCT03986528) on May 30, 2019. Recruitment began in August 28, 2019. Expected date when recruitment will be completed in December, 2020. Due to COVID-19, the recruitment is expected to end in December 2021. Analysis of the primary outcome measure will be completed in March, 2022. The study will end in May, 2022.

## Data Availability

The results of the study will be issued to publications through scientific journals and conference reports. The anonymized datasets used and/or analyzed during the current study are available from the corresponding author on reasonable request.

## Abbreviations

NSCLC: Non-small-cell lung cancer
KLTi: Kanglaite injections
TCM: Traditional Chinese medicine
PFS: Progression-free survival
OS: Overall survival
ORR: Objective response rate
RECIST 1.1: The Response Evaluation Criteria in Solid Tumors 1.1
ECOG: Eastern Cooperative Oncology Group (ECOG)
EGFR: Epidermal growth factor receptor
ALK: Activin receptor-like kinase
ROS1: c-ros oncogene 1
PD-L1: Programmed death-ligand 1
NCCN: National Comprehensive Cancer Network
CR: Complete response
PR: Partial response
EORTC: European Organization for Research and Treatment of Cancer
QLQ-C30: Quality of Life Questionnaire Core 30
LCSS: Lung Cancer Symptom Scale
KPS: Karnofsky performance status
TC: Total cholesterol
TG: Triglyceride
HDL-C: High-density lipoprotein cholesterol
LDL-C: Low-density lipoprotein cholesterol
AEs: Adverse events
CTCAE v4.03: National Cancer Institute’s Common Terminology Criteria for Adverse Events v4.03
EMEA: European Medicines Agency
FDA: Food and Drug Administration
